# The antihyperlipidemic activities of 4(3H) quinazolinone and two halogenated derivatives in rats

**DOI:** 10.1186/1476-511X-4-22

**Published:** 2005-10-04

**Authors:** Fawzia M Refaie, Amr Y Esmat, Soad M Abdel Gawad, Aida M Ibrahim, Mona A Mohamed

**Affiliations:** 1 Department of Biochemistry, Faculty of Science, Ain Shams University, Cairo, Egypt; 2 Department of Organic Chemistry, Faculty of Science, Al-Azhar University, Cairo, Egypt; 3 Department of Clinical Pathology, Faculty of Medicine, Al-Azhar University, Cairo, Egypt

**Keywords:** Quinazolinone, Halogenated quinazolinones, Bezafibrate, hypercholesterolemia, Diabetes mellitus, Triglycerides, Cholesterol, lipoproteins, Rat

## Abstract

In the present study, the effects of subchronic treatments (4 weeks) of hypercholesterolemic (single) and diabetic-hypercholesterolemic (combined) rats with 4 (3H) quinazolinone and 2 halogenated derivatives (6, 8-dibromo-2-methy-4 (3H) quinazolinone and 6-iodo-2-methyl-4(3H) quinazolinone) at a sublethal dose level (2 mg/Kg) on cholesterol metabolism were investigated. Bezafibrate, a hypolipidemic drug was used as a reference compound for data comparison. Treatment of rats with single and combined hypercholesterolemia with quinazolinone compounds gave rise to highly significant reductions in serum total cholesterol and cholesterol ester levels, whereas serum triacylglycerol level was significantly reduced only after treatment with halogen-substituted quinazolinones in single hyper-cholesterolemia, compared to the control group. The effects of different quinazolinones and bezafibrate on reduction of serum LDL-C level were comparable in single hypercholesterolemia but significantly different in combined hypercholesterolemia. Results obtained from this study suggest that the antihyperlipidemic effect of quinazolinone compounds was brought about by inhibition of dietary cholesterol absorption and / or intestinal ACAT activity.

## Introduction

Cardiovascular diseases remain by far the number one cause of death for both men and women of all ethnic backgrounds. Although many causative factors of these diseases are recognized (smoking, high blood pressure, genetic background, diabetes mellitus and obesity) high serum LDL-C and elevated total cholesterol levels are the most prevalent indicators for susceptibility to atherosclerotic heart disease [1,2]. Atherosclerosis is a disorder of the arterial wall characterized by accumulation of cholesterol ester in cells derived from the monocyte-macrophage line, smooth muscle cell proliferation and fibrosis, and results in narrowing the blood vessel [3]. An association of dietary cholesterol with cardiac and cerebral vascular diseases is based on several lines of evidence, including studies in animal models and epidemiological data in humans [4].

Dyslipidemia, hallmarked by low plasma HDL-C and high LDL-C and triacylglycerol levels, is common in patients with diabetes mellitus. These lipoprotein abnormalities are held to be responsible for considerable cardiovascular disease-related morbidity and mortality [5]. The risk for cardiovascular disease is increased approximately 2 to 4 fold in patients with diabetes mellitus compared with non-diabetic controls [6].

There are many classes of lipid lowering agents available, these drugs have different mechanisms of action and variable efficacy depending on the lipid profile of an individual. In spite of their lipid-lowering effect, these drugs have many side effects. Thus, research is still pursuing to find out novel agents that are more effective and safe. The 4(3H)-quinazolinone derivatives (a derivative of the parent compound quinazoline) have been shown as a group of compounds of broad medical interest. Quinazolinones are reported to exhibit antibacterial [7], antiviral [8], anticancer [9], antihypertensive [10] and anti-inflammatory activities [11]. The antihyperlipidemic and antihypercholesterolemic activities of quinazolinone derivatives are reported and the activities of the tested compounds were almost equal to that of β-sitosterol (a plant sterol of hypolipidemic activity) [1,12].

The present work aims at investigating the antihyperlipidemic activities of 4 (3H) quinazolinone and two halogenated derivatives (6, 8-dibromo- and 6-iodo-2-methyl-4(3H) quinazolinone) in hypercholesterolemic and/or diabetic rats. Bezafibrate was adopted in this study as a reference hypolipidemic drug for data comparison.

## Materials and methods

### Chemicals

4(3H)-quinazolinone was synthesized by stirring a mixture of formamide and anthranilic acid at 120–125°C for 4 h. [7]. The two halogenated derivatives; 6, 8-dibromo-2-methyl-4(3H)-quinazolinone and 6-iodo-2-methyl-4(3H)-quinazolinone were synthesized as previously described [13]. The crude compounds were filtered, washed several times with hot water then recrystallized from ethanol. The structures of the synthesized compounds were verified by IR and mass spectral analyses.

### Animals

A total of 130 adult male Swiss albino rats weighing 150–180 g were used throughout this study. The animals were housed in steel mesh cages (4/cage) and maintained for a week-acclimatization period on a commercial pellet diet, which was finely ground before being administered to animals.

### Preparation and Administration of Chemical Compounds

One tablet (200 mg) of the bezafibrate (trivial name: Bezalip, GlaxoWellcome, UK) was finely ground and dissolved in 50 ml of distilled water to prepare a stock solution of 4 mg/ml. The solution was prepared weekly and stored in a well stoppered bottle at 4°C. Bezafibrate was daily administered to rats by a stainless-steel gavage needle at a dose level of 18 mg/kg body weight, which is equivalent to the human dose calculated from the conversion table of Paget and Barnes [14].

A weight of 50 mg quinazolinone and its halogenated derivatives was dissolved in 50 ml of 10% (v/v) polyethylene glycol to prepare stock solutions (1 mg/ml). The solutions were prepared weekly and stored in a well-stoppered bottle at 4°C. The quinazolinone compounds were orally administered to animals at a sublethal dose level of 2 mg/kg body weight. The LD_50 _value of quinazolinone was previously reported to be 320 mg/kg body weight on i.p. administration in rats [15]. The toxicities of dibromo-and iodoquinazolinone derivatives were very low as they did not cause any mortality of rats in excess of 800 mg/kg body weight.

### Preparation of High Cholesterol Diet

The high cholesterol diet was prepared as previously described [16]. 1% cholesterol (w/w) and 0.2% sodium cholate (w/w) were well mixed with the finely-ground commercial diet.

### Induction of Diabetes Mellitus

Induction of diabetes mellitus in rats was carried out by a single i.p. injection of freshly prepared alloxan monohydrate (Sigma, USA) solution at a concentration of 120 mg/kg body weight after a fast of 12 h. [17]. After 3 days of diabetes mellitus induction rats were put on the high cholesterol diet for 4 weeks.

### Study Design

Rats were allocated into 4 main reference groups (I-IV) as follows: Group I. Normal Controls (NC) comprised 8 normal rats fed a normal diet and left intact without any treatment, Group II. Vehicle Controls consisted of 8 normal rats fed a normal diet and received daily an oral dose of 0.5 ml 10% polyethylene glycol, Group III. Hypercholesterolemia, which was subdivided into 5 subgroups (8 rats each): (a) Hypercholesterolemia Controls, in which rats fed a high cholesterol diet and left without any treatment and (b, c, d, e) subgroups, which consisted of hypercholesterolemic rats treated daily with a single oral dose of bezafibrate, 4 (3H) quinazolinone, 6, 8-dibromo or 6-iodo-2-methyl-4 (3H) quinazolinone, respectively, and Group IV. Combined Hypercholesterolemia (C), which was also subdivided into 5 subgroups (8 rats each): (a) Diabetes- Hypercholesterolemia controls, in which diabetic rats fed a high cholesterol diet and left without any treatment and (b, c, d, e) subgroups, in which diabetic rats fed a high cholesterol diet and treated daily with a single oral dose of bezafibrate, 4 (3H) quinazolinone, 6, 8-dibromo or 6-iodo-2-methyl-4 (3H) quinazolinone, respectively.

Treatment with the different compounds started on the same day of feeding high cholesterol diet and lasted for 4 weeks. Body weight of the animals in all groups was recorded weekly until the end of the experiment.

### Blood Collection and Tissue Sampling

Blood samples were taken from the retro-orbital venous plexus under light ether anesthesia using a glass capillary tube after a fast of 12 h. and immediately centrifuged. Serum samples were aliquoted and stored at -20°C until lipid profile analysis, except for fasting glucose, LDL and HDL levels, which were determined on the same day without delay. Liver was excised, rinsed from blood in isotonic sterile saline, blotted dry and weighed. A weighed portion of fresh liver tissue was dropped into a test tube containing concentrated sulphuric acid for total lipid determination. The remainder of liver tissue was stored in physiological saline at -20°C until biochemical analysis.

### Biochemical Assays

Fasting serum glucose level was determined by an enzymatic colorimetric method [18] using a commercial assay kit (Diamond Diagnostics, Egypt). Serum and hepatic total cholesterol and cholesterol ester levels were measured according to Zlatkis *et al*. [19]. Serum triacylglycerol level was assayed by the method of Jacobs and Van Denmark [20] (Diamond Diagnostics, Egypt). Serum and hepatic total lipid levels were determined as described by Knight *et al*. [21]. Serum HDL-C [22] and LDL-C levels [23] were also assessed (Diamond Diagnostics, Egypt and Quimica Clinica Aplicada, Spain, respectively). Serum VLDL-C level and the atherogenic index were determined by calculation [24,25]. Liver and kidney function tests (serum albumin, urea, creatinine, alanine and aspartate aminotransferases activity) were determined using commercial assay kits (Diamond Diagnostics, Egypt). The concentrations of triacylglycerol and malondialdehyde were also estimated in the whole liver homogenate (5%) [26,27].

### Statistical Analysis

Data were expressed as means ± SD and the least significant difference (LSD) test for multiple comparisons between the different treated subgroups and along with their respective controls was applied [28].

## Results

**Figure 1:**
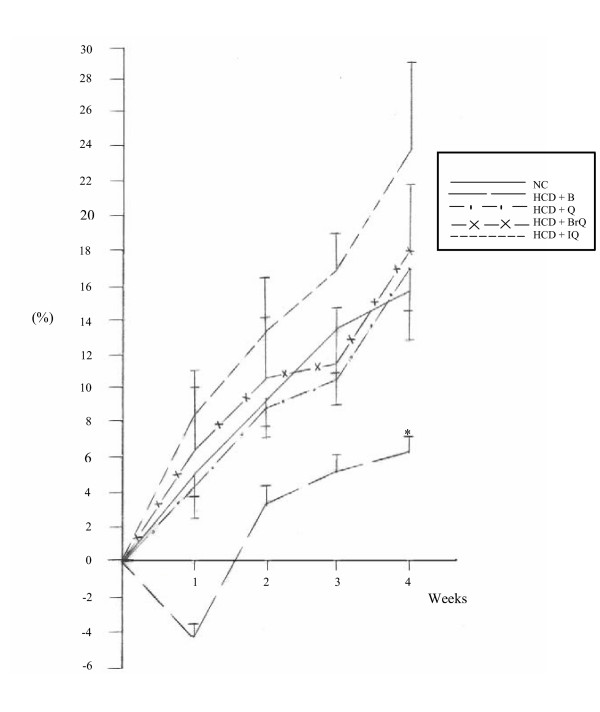
Changes in the percentage of body weight gain in hypercholesterolemic rats treated with the different modalities throughout the experimental period. *denotes statistical significance (p < 0.05) at the 4^th ^week point compared to normal controls.

Fig. 1 shows a gradual increase in the percentage of total body weight gain in hypercholesterolemic rats treated with bezafibrate, quinazolinone and its halogenated derivatives throughout the experimental period. Non significant changes were recorded in the percentage of body weight gain in the treated subgroups at the 4^th ^week point, except for the bezafibrate-treated subgroup, which showed a significant decrease compared to normal controls.

**Figure 2:**
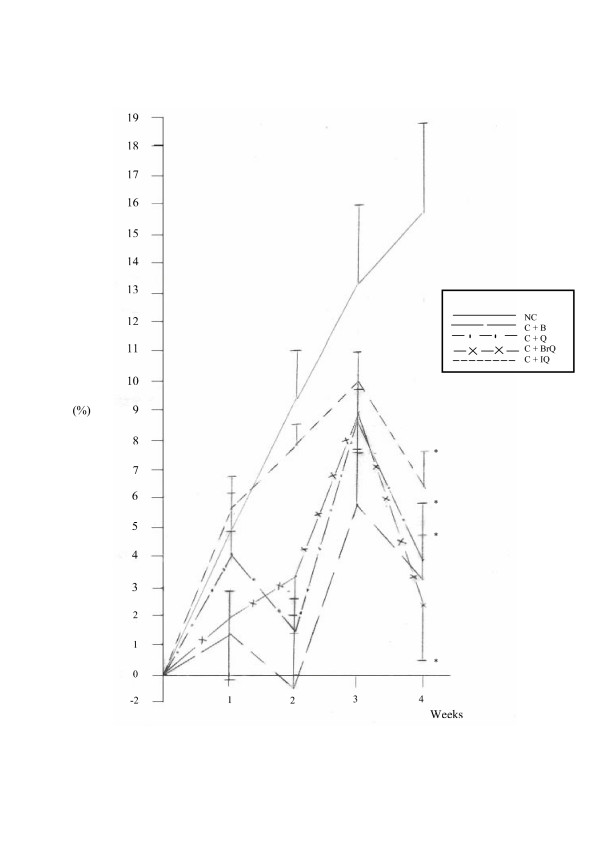
Changes in the percentage of body weight gain in diabetic-hypercholesterolemic rats treated with the different modalities throughout the experimental period. *denotes statistical significance (p < 0.05) at the 4^th ^week point compared to normal controls.

Fig. 2 demonstrates irregular changes in the percentage of body weight gain in diabetic-hypercholesterolemic rats treated with bezafibrate, quinazolinone and its halogenated derivatives culminated in a highly significant reduction at the end of the experiment, which reached 79.32, 74.92, 85.32 and 59.48% (P < 0.01), respectively, compared to normal controls.

Table 1 illustrates non significant differences in relative liver weights in both non diabetic and diabetic-hypercholesterolemic groups treated with the different compounds.

**Table 1:** Effects of bezafibrate, quinazolinone and its halogenated derivatives on relative liver weights of rats with single and combined hypercholesterolemia compared to normal controls.

**Single Hypercholesterolemia**		**Combined Hypercholesterolemia**	
**Groups**	Liver wt./b.wt.	**Groups**	Liver wt./b.wt.

N.C		N.C	
Mean ± SD	0.031 ± 0.005	Mean ± SD	0.031 ± 0.005
Range	(0.024–0.039)	Range	(0.024–0.039)
HCD+B		C+B	
Mean ± SD	0.030 ± 0.003	Mean ± SD	0.037 ± 0.005
Range	(0.025–0.032)	Range	(0.032–0.045)
Change%	-11.76	Change%	5.71
HCD+Q		C+Q	
Mean ± SD	0.033 ± 0.003	Mean ± SD	0.030 ± 0.002
Range	(0.029–0.037)	Range	(0.028–0.033)
Change%	-2.94	Change%	-14.29
HCD+BrQ		C+BrQ	
Mean ± SD	0.031 ± 0.003	Mean ± SD	0.029 ± 0.003
Range	(0.028–0.035)	Range	(0.025–0.036)
Change%	-8.82	Change%	-17.14
HCD+IQ		C+IQ	
Mean ± SD	0.030 ± 0.002	Mean ± SD	0.030 ± 0.001
Range	(0.027–0.032)	Range	(0.027–0.031)
Change%	-11.76	Change%	-14.29

Table 2 shows that treatment of hypercholesterolemic rats with bezafibrate, quinazolinone, dibromo- and iodo-derivatives caused a significant reduction in fasting serum glucose level, which reached 11.63, 29.78, 22.47 and 29.40%, respectively, compared to the control group. Multiple comparison analysis showed that quinazolinone and its halogenated derivatives produced a more pronounced antihyperglycemic effect than bezafibrate. Bezafibrate and the three quinazolinone compounds gave rise to a comparable decrease of 18.12, 27.73, 31.66 and 32.31%, respectively, in serum total lipids concentration. A parallel reduction in serum triacylglycerol level was noticed in bezafibrate (26.65%), dibromo- (16.83%) and iodoquinazolinone (15.74%) subgroups, compared to the control group. Treatment with bezafibrate, quinazolinone and its dibromo-derivative caused a comparable reduction in serum total cholesterol and cholesterol ester levels, which reached 28.78 & 37.7, 39.93 & 47.77 and 41.41 & 46.85%, respectively. Iodoquinazolinone showed a more notable decrease of 43.83 and 50.78%, respectively. Serum free cholesterol and HDL-C levels were insignificantly changed in all treatment subgroups. In contrast, significantly parallel reductions in serum LDL-C level and atherogenic index were recorded in all treated subgroups, and reached 38.56 & 41.29, 55.03 & 56.73, 55.65 & 49.91 and 54.44 & 37.70% in bezafibrate, quinazolinone, dibromo- and iodoquinazolinone, respectively. Serum VLDL-C level was significantly reduced in bezafibrate (26.63%), dibromo- (16.83%) and iodoquinazolinone (15.72%) subgroups, compared to controls. Multiple comparison analysis showed non significant changes in serum VLDL-C level among the different treatment subgroups.

**Table 2:** Statistical significance of fasting serum glucose level and lipid profile in hypercholesterolemic rats treated with bezafibrate, quinazolinone and halogenated derivatives, compared to control group.

**Parameters**	**HCD**	**HCD+B**	**HCD+Q**	**HCD+BrQ**	**HCD+IQ**
Glucose(mg/dl)					
Mean ± SD	123.57 ± 11.99^a^	109.20 ± 14.31^b^	86.77 ± 9.87^c^	95.80 ± 8.28^c^	87.24 ± 5.32^c^
Range	(105.11–135.02)	(82.69–125.22)	(77.45–106.22)	(86.36–111.10)	(77.54–93.81)
Change%	-	-11.63	-29.78	-22.47	-29.40
Total lipids(g/L)					
Mean ± SD	4.58 ± 0.67^a^	3.75 ± 0.68^b^	3.31 ± 0.95^b^	3.13 ± 0.43^b^	3.10 ± 0.78^b^
Range	(3.62–5.67)	(2.82–4.79)	(2.02–4.54)	(2.40–3.90)	(2.17–4.72)
Change%	-	-18.12	-27.73	-31.66	-32.31
T.G.(mg/dl)					
Mean ± SD	94.46 ± 14.23^a^	69.29 ± 11.04^b^	87.05 ± 9.18^ac^	78.56 ± 12.79^bc^	79.59 ± 15.94^bc^
Range	(73.33–108.89)	(54.40–86.65)	(75.56–102.22)	(66.67–98.50)	(52.44–100.89)
Change%	-	-26.65	-7.84	-16.83	-15.74
T.Cholesterol(mg/dl)					
Mean ± SD	159.06 ± 26.04^a^	113.28 ± 17.52^b^	95.55 ± 18.11^bc^	93.19 ± 14.29^bc^	89.35 ± 22.37^c^
Range	(119.35–87.50)	(95.93–139.06)	(75.63–121.41)	(65.63–104.69)	(70.31–123.44)
Change%	-	-28.78	-39.93	-41.41	-43.83
F.Cholesterol(mg/dl)					
Mean ± SD	36.66 ± 7.14^a^	37.88 ± 8.94^a^	32.12 ± 8.15^a^	29.45 ± 4.11^a^	30.14 ± 6.39^a^
Range	(25.76–45.20)	(29.59–52.36)	(21.14–46.18)	(22.11–33.82)	(25.04–40.33)
Change%	-	3.33	-12.38	-19.67	-17.79
E.Cholesterol(mg/dl)					
Mean ± SD	122.40 ± 19.10^a^	76.25 ± 8.89^b^	63.93 ± 10.02^bc^	65.05 ± 7.96^bc^	60.25 ± 15.29^c^
Range	(91.85–142.30)	(66.01–89.04)	(50.54–76.01)	(54.00–74.33)	(43.32–83.11)
Change%	-	-37.7	-47.77	-46.85	-50.78
HDL-C (mg/dl)					
Mean ± SD	24.27 ± 3.23 ^a^	27.73 ± 5.48^a^	28.33 ± 6.15^a^	25.68 ± 6.34^a^	21.14 ± 8.93^a^
Range	(19.75–28.80)	(18.30–35.15)	(20.36–36.00)	(18.92–33.32)	(14.44–37.84)
Change%	-	14.26	16.73	5.81	-12.90
LDL-C (mg/dl)					
Mean ± SD	116.09 ± 26.74^a^	71.32 ± 17.78 ^b^	52.20 ± 15.23^b^	51.49 ± 10.43 ^b^	52.89 ± 19.31^b^
Range	(74.82–141.75)	(49.96–89.66)	(32.83–76.84)	(29.54–62.17)	(26.84–79.28)
Change%	-	-38.56	-55.03	-55.65	-54.44
VLDL-C (mg/dl)					
Mean ± SD	18.89 ± 2.85^a^	13.86 ± 2.21^b^	17.41 ± 1.84^ac^	15.71 ± 2.56^bc^	15.92 ± 3.19^bc^
Range	(14.67–21.78)	(10.88–17.33)	(15.11–20.44)	(13.33–19.70)	(10.49–20.18)
Change%	-	-26.63	-7.83	-16.83	-15.72
A.I.					
Mean ± SD	5.57 ± 0.87^a^	3.27 ± 1.30^b^	2.41 ± 0.42^b^	2.79 ± 1.02^b^	3.47 ± 0.91^b^
Range	(4.22–7.21)	(1.80–6.11)	(1.94–3.14)	(1.67–4.52)	(2.26–4.70)
Change%	-	-41.29	-56.73	-49.91	-37.70

For each parameter similar characters denote insignificance between groups. The mean difference is significant at p < 0.05.

Table 3 illustrates that treatment of diabetic-hypercholesterolemic rats with bezafibrate caused significant reductions in fasting serum glucose and total lipid levels (17.36 and 23.84%, respectively), while more pronounced reductions were recorded after treatment with quinazolinone (67.48 & 48.32%), dibromo- (66.09 & 49.76%) and iodo- derivatives (67.39 & 47.52%), compared to the control group. Significant decreases were recorded in serum levels of total cholesterol and cholesterol ester levels after treatment with quinazolinone (43.66 & 62.33%), dibromo- (49.86 & 55.34%) and iodo-derivative (49.12 & 56.74%, respectively), whereas bezafibrate caused a less notable decrease of serum cholesterol ester level (19.93%) and did not affect total cholesterol level. None of the foregoing treatments significantly affected serum triacylglycerol, HDL-C and VLDL-C levels. Treatment of diabetic-hypercholesterolemic rats with quinazolinone, dibromo- and iodo- derivatives produced significant decreases in serum LDL-C level (71.74, 65.69 and 60.74%, respectively) and atherogenic index (50.24, 69.54 and 51.99%). Bezafibrate treatment caused a slight but significant reduction in the atherogenic index (28.39%), while serum LDL-C level remains unchanged.

**Table 3:** Statistical significance of serum fasting glucose level and lipid profile in diabetic-hypercholesterolemic rats treated with bezafibrate, quinazolinone and halogenated derivatives.

**Parameters**	**C**	**C+B**	**C+Q**	**C+BrQ**	**C+IQ**
Glucose(mg/dl)					
Mean ± SD	210.34 ± 16.56^a^	173.82 ± 26.86^b^	68.40 ± 5.22^c^	71.32 ± 8.42^c^	68.60 ± 5.66^c^
Range	(189.24–230.00)	(140.00–218.00)	(61.65–79.47)	(60.55–85.00)	(60.76–75.36)
Change%	-	-17.36	-67.48	-66.09	-67.39
Total lipids(g/L)					
Mean ± SD	6.25 ± 2.29^a^	4.76 ± 0.85^b^	3.23 ± 1.08^c^	3.14 ± 0.63^c^	3.28 ± 0.77^c^
Range	(2.32–9.51)	(3.76–5.96)	(2.02–4.64)	(2.02–4.15)	(2.20–4.34)
Change%	-	-23.84	-48.32	-49.76	-47.52
T.G(mg/dl)					
Mean ± SD	110.42 ± 18.75^a^	91.91 ± 15.64^a^	109.51 ± 31.98^a^	99.00 ± 15.06^a^	100.64 ± 10.33^a^
Range	(88.14–133.33)	(70.00–111.11)	(66.67–165.00)	(73.33–121.14)	(86.67–117.11)
Change%	-	-16.76	-0.82	-10.34	-9.72
T.Cholesterol(mg/dl)					
Mean ± SD	176.28 ± 42.14^a^	152.76 ± 36.36^a^	99.31 ± 22.80^b^	88.38 ± 26.64^b^	89.69 ± 10.02^b^
Range	(127.34–224.38)	(106.25–199.22)	(74.22–126.56)	(65.63–134.38)	(73.44–104.22)
Change%	-	-13.34	-43.66	-49.86	-49.12
F.Cholesterol(mg/dl)					
Mean ± SD	39.19 ± 11.02^ab^	44.37 ± 9.44^a^	30.31 ± 6.98^b^	30.03 ± 6.73^b^	31.06 ± 5.43^b^
Range	(25.62–51.89)	(28.62–53.98)	(20.16–40.98)	(23.09–41.30)	(26.02–41.63)
Change%	-	13.22	-22.66	-23.37	-20.75
E.Cholesterol(mg/dl)					
Mean ± SD	135.37 ± 31.41^a^	108.39 ± 31.2^b^	69.00 ± 18.80^c^	60.46 ± 16.43^c^	58.56 ± 6.96^c^
Range	(101.72–170.75)	(77.63–145.24)	(49.18–96.97)	(48.40–88.08)	(47.42–65.67)
Change%	-	-19.93	-62.33	-55.34	-56.74
HDL-C(mg/dl)					
Mean ± SD	24.76 ± 3.62^a^	25.88 ± 2.74^a^	24.87 ± 4.71^a^	26.71 ± 2.58^a^	22.50 ± 1.77^a^
Range	(18.00–27.94)	(21.13–29.60)	(19.83–31.52)	(22.40–30.33)	(19.80–24.32)
Change%	-	4.52	0.44	7.88	-9.13
LDL-C(mg/dl)					
Mean ± SD	115.29 ± 34.06^a^	84.56 ± 43.44^a^	32.58 ± 18.85^b^	39.56 ± 26.65^b^	45.26 ± 10.16^b^
Range	(79.73–168.69)	(44.05–137.56)	(19.51–64.94)	(16.24–82.42)	(34.31–63.03)
Change%	-	-26.65	-71.74	-65.69	-60.74
VLDL-C(mg/dl)					
Mean ± SD	22.08 ± 3.75^a^	18.38 ± 3.13^a^	22.52 ± 6.28^a^	19.80 ± 3.01^a^	20.13 ± 2.07^a^
Range	(17.63–26.67)	(14.00–22.22)	(13.33–33.00)	(14.67–24.23)	(17.33–23.42)
Change%	-	-1.67	1.99	-10.33	-8.83
A.I.					
Mean ± SD	6.27 ± 2.06^a^	4.49 ± 1.53^b^	3.12 ± 1.20^b^	1.91 ± 1.27^b^	3.01 ± 0.57^b^
Range	(3.56–9.80)	(2.59–6.91)	(1.65–4.82)	(0.99–4.33)	(2.21–3.79)
Change%	-	-28.39	-50.24	-69.54	-51.99

For each parameter similar characters denote insignificance between groups.

The mean difference is significant at p < 0.05.

Table 4 demonstrates that treatment of hypercholesterolemic rats with bezafibrate, quinazolinone, dibromo- and iodo- derivatives caused a significant parallel reduction in hepatic total lipids concentration (20.11, 30.80, 25.01 and 25.21%, respectively) associated with a significant comparable elevation in hepatic malondialdehyde concentration (74.95, 63.11, 49.84 and 60.81%), compared to the control group. Hepatic triacylglycerol concentration was significantly decreased in bezafibrate (43.03%), dibromo- (25.59%) and iodoquinazolinone (23.70%) subgroups, while it remains unchanged in quinazolinone subgroup, compared to the control group. A significant elevation in hepatic free cholesterol and, in contrast a significant decrease of hepatic cholesterol ester concentrations were recorded in quinazolinone (120.34 & 50.67%, respectively), dibromo-(66.21 & 32.09%) and iodoquinazolinone (63.28 & 31.06%) subgroups. The LSD test revealed that halogenated quinazolinones exerted a more pronounced effect on cholesterol fractions than quinazolinone. Bezafibrate produced no significant effect on hepatic cholesterol fractions. Also, hepatic total cholesterol concentration was not significantly changed in all treatment subgroups.

**Table 4:** Statistical significance of hepatic lipid profile and malondialdehyde concentration in hypercholesterolemic rats treated with bezafibrate, quinazolinone and halogenated derivatives, compared to control group

**Parameters**	**HCD**	**HCD+B**	**HCD+Q**	**HCD+BrQ**	**HCD+IQ**
Total lipids					
Mean ± SD	75.30 ± 7.86^a^	60.16 ± 4.18^b^	52.11 ± 13.07^b^	56.47 ± 9.50^b^	56.32 ± 12.94^b^
Range	(68.24–88.24)	(54.90–66.27)	(36.86–74.90)	(41.57–68.24)	(41.57–76.08)
Change%	-	-20.11	-30.80	-25.01	-25.21
Triacylglycerol					
Mean ± SD	10.55 ± 2.66^a^	6.01 ± 1.39^b^	9.83 ± 2.17^a^	7.85 ± 2.75^b^	8.05 ± 1.35^b^
Range	(7.09–14.91)	(4.09–7.83)	(7.30–13.91)	(4.39–11.74)	(6.65–10.50)
Change%	-	-43.03	-6.82	-25.59	-23.70
Total cholesterol					
Mean ± SD	25.18 ± 2.77^ab^	25.64 ± 1.48^a^	22.71 ± 2.13^b^	22.80 ± 2.01^b^	22.83 ± 2.13^b^
Range	(20.58–28.46)	(23.98–28.32)	(19.11–25.85)	(20.00–25.39)	(19.12–25.61)
Change%	-	1.83	-9.81	-9.45	-9.33
Free cholesterol					
Mean ± SD	5.80 ± 1.70^a^	5.23 ± 1.32^a^	12.78 ± 2.46^b^	9.64 ± 1.30^c^	9.47 ± 1.50^c^
Range	(3.37–8.13)	(3.85–6.83)	(9.52–17.33)	(7.68–11.45)	(7.22–11.51)
Change%	-	-9.83	120.34	66.21	63.28
Ester cholesterol					
Mean ± SD	19.38 ± 1.67^a^	20.42 ± 1.00^a^	9.56 ± 1.56^b^	13.16 ± 1.30^c^	13.36 ± 1.20^c^
Range	(16.99–21.76)	(19.35–22.47)	(7.57–11.43)	(11.11–14.51)	(11.67–15.56)
Change%	-	5.37	-50.67	-32.09	-31.06
MDA(nmol/g)					
Mean ± SD	131.31 ± 15.10^a^	229.73 ± 4.76^b^	214.18 ± 23.85^b^	196.75 ± 26.44^b^	211.16 ± 14.54^b^
Range	(113.33–155.42)	(223.53–238.43)	(172.42–246.01)	(161.57–229.28)	(188.37–230.33)
Change%	-	74.95	63.11	49.84	60.81

All parameters are expressed in mg/g tissue unless otherwise stated.

For each parameter similar characters denote insignificance between groups.

The mean difference is significant at p < 0.05.

Table 5 shows that hepatic total lipids, triacylglycerol, total cholesterol and malondialdehyde concentrations were not significantly changed in diabetic-hypercholesterolemic rats treated with quinazolinone and its halogenated derivatives. A significant parallel elevation in hepatic free cholesterol and, in contrast a significant parallel decrease of hepatic cholesterol ester concentrations were recorded in quinazolinone (65.53 & 32.62%, respectively), dibromo-(68.94 & 30.48%) and iodoquinazolinone subgroups (67.44 & 25.59%). Bezafibrate treatment had no significant effect on all of the foregoing parameters.

**Table 5:** Statistical significance of hepatic lipid profile and malondialdehyde concentration in diabetic-hypercholesterolemic rats treated with bezafibrate, quinazolinone and halogenated derivatives

**Parameters**	**C**	**C+B**	**C+Q**	**C+BrQ**	**C+IQ**
Total lipids					
Mean ± SD	74.76 ± 7.34^a^	65.00 ± 7.37^a^	71.37 ± 6.01^a^	65.93 ± 11.54^a^	66.12 ± 9.47^a^
Range	(65.88–84.71)	(54.90–76.47)	(62.16–77.65)	(45.49–81.57)	(48.92–78.43)
Change%	-	-13.06	-4.53	-11.81	-11.56
Triacylglycerol					
Mean ± SD	12.10 ± 3.37^a^	9.90 ± 1.08^a^	11.90 ± 4.38^a^	12.03 ± 5.07^a^	11.80 ± 4.82^a^
Range	(8.87–17.30)	(8.38–11.30)	(7.48–16.61)	(6.09–18.78)	(6.23–17.30)
Change%	-	-18.18	-1.65	-0.58	-2.48
Total cholesterol					
Mean ± SD	26.53 ± 4.93^a^	26.58 ± 3.37^a^	23.87 ± 3.58^a^	25.62 ± 2.94^a^	26.57 ± 2.88^a^
Range	(20.05–34.15)	(21.14–31.71)	(18.79–28.86)	(23.58–32.52)	(22.08–30.08)
Change%	-	0.19	-10.03	-3.43	0.15
Free cholesterol					
Mean ± SD	7.34 ± 2.45^a^	7.49 ± 1.93^a^	12.15 ± 2.42^b^	12.40 ± 3.17^b^	12.29 ± 2.59^b^
Range	(4.04–10.41)	(5.69–11.32)	(8.29–16.03)	(8.85–18.31)	(8.98–14.80)
Change%	-	2.04	65.53	68.94	67.44
Cholesterol ester					
Mean ± SD	19.19 ± 2.61^a^	19.19 ± 1.95^a^	12.93 ± 2.78^b^	13.34 ± 2.77^b^	14.28 ± 3.82^b^
Range	(15.31–23.74)	(16.26–21.93)	(9.25–16.69)	(10.36–18.21)	(9.57–19.02)
Change%	-	0	-32.62	-30.48	-25.59
MDA(nmol/g)					
Mean ± SD	235.18 ± 16.32^a^	238.29 ± 18.23^a^	250.98 ± 12.06^a^	250.23 ± 13.03^a^	244.19 ± 13.45^a^
Range	(210.72–256.47)	(218.99–269.02)	(234.51–267.97)	(237.65–276.24)	(221.17–257.91)
Change%	-	1.32	6.72	6.40	3.83

All parameters are expressed in mg/g tissue unless otherwise stated.

For each parameter similar characters denote insignificance between groups.

The mean difference is significant at p < 0.05.

## Discussion

Although the literature implies a few evidence about the antihyperlipidemic activity of some quinazolinone derivatives, yet intensive research in quinazolinone chemistry is still in progress as indicated by the continuous flow of newly appearing derivatives. In the present study, we have demonstrated the effects of quinazolinone and two halogenated derivatives on the lipid profile in single and combined hypercholesterolemia in rats. Alloxan was used to induce diabetes mellitus in rats and, surprisingly treatment of single and combined hypercholesterolemia groups with quinazolinone compounds for 4 weeks significantly decreased the fasting serum glucose level, compared to bezafibrate and their respective control groups (Tables 2 &3). Although the antihyperglycemic activity of these compounds was not intentionally studied, this effect could be attributed to being cyclic amidine compounds. Metformin, a well known hypoglycemic drug, which acts as an inhibitor of hepatic glucose production, possesses guanidine and amidine functionalities in its molecular structure. Another class of compounds, triarylimidazoles, which has also an amidine moiety in a cyclic structure, displayed a significant glucagon antagonistic property [29]. The antihyperglycemic activity of the quinazolinone compounds is worth to be adequately investigated.

The quinazolinone compounds, being water insoluble, were dissolved in 10% polyethylene glycol as a solvent and thus a vehicle control group was established to evaluate its effect on all studied serum and liver tissue parameters. Statistical analysis showed that polyethylene glycol had no significant effect on the body weight of animals, as well as all serum and liver tissue parameters (data not shown). Accordingly, statistical significance was referred to the normal control or the reference groups.

In order to assess the toxic side effects of the administered dose levels of quinazolinone compounds, the change percent of body weight gain at each week point with respect to the initial body weight was recorded. In comparison to normal controls at the 4^th ^week point, hypercholesterolemic rats treated with quinazolinone compounds exhibited non significant changes in the percentage of body weight gain, except for bezafibrate subgroup, which showed a significant reduction (P < 0.01) (Fig. 1). Diabetic-hypercholesterolemic rats manifested a significant decrease in the percentage of body weight gain (P < 0.01) in the different treatment modalities (Fig. 2). This clearly indicates that quinazolinone compounds had no significant effect on body weight, and that the reduction in the body weight gain of diabetic- hypercholesterolemic animals was mainly due to diabetes mellitus. Sellei *et al*. [30] reported that treatment with a therapeutic dose of any compound on the body weight loss must not exceed 10%. Also, Bissery *et al*. [31] stated that a drug dosage that produces a loss in body weight of 20% is considered as excessively toxic. Feron *et al*. [32] documented that in subchronic toxicity experiments, the weights of the major organs of the body may serve as a useful index of toxicity. However, decreased absolute weights in treated animals may be merely a reflection of lower body weight, thus calculation of organ weight to whole body weight ratio justifies the usefulness of the data obtained. In the present study, relative liver weights were recorded, as liver is the main site of drug activation and detoxification. Non significant differences in relative liver weights were observed in single and combined hypercholesterolemia subgroups (Table 1). Also, non significant alterations were recorded in serum levels of aminotransferases activity (ALT&AST) and albumin, as well as urea and creatinine concentrations, compared to normal controls (data not shown), which indicates the safety of the quinazolinone compounds at the adopted dose levels.

A comparable reduction in serum and hepatic triacylglycerol levels is reported in hypercholesterolemic rats treated with bezafibrate, dibromo- and iodoquinazolinone subgroups, compared to their reference groups (Tables 2 &4), which demonstrates that halogens substitution in the quinazolinone nucleus confered a triacylglycerol-lowering effect to the parent quinazolinone compound. However, this observation was not noticed in combined hypercholesterolemia (Tables 3 &5) and may be interpreted as simply due to increased fat mobilization as a result of insulin deficiency manifested by the significant reduction in the body weight of the animals at decapitation (Fig. 2).

The cholesterol-lowering effect of quinazolinone and its dibromo-and iodo-derivatives is represented by comparable reductions in serum total cholesterol and cholesterol ester levels in single and combined hypercholesterolemia (Tables 2 &3), which might be due to inhibition of dietary cholesterol absorption and/or its esterification. Since two enzymes are involved in these two processes; pancreatic cholesterol esterase [33] and intestinal acyl CoA-cholesterol acyl transferase enzyme (ACAT) [34], thus it could be suggested that quinazolinones inhibit one or both enzymes activity. The assumption that quinazolinones inhibit ACAT activity is confirmed by the significant decrease of hepatic cholesterol ester concentration and, in contrast the significant increase of hepatic free cholesterol concentration (Tables 4 &5). The remarkable elevation in hepatic MDA concentration in single hypercholesterolemia after treatment with quinazolinones, compared to their reference group (Table 4) may be explained due to increased fatty acids pool and consequently its peroxidation as a result of ACAT inhibition by quinazolinone compounds (as previously mentioned). Another suggestion for the cholesterol-lowering effect of the quinazolinones is the interaction between quinazolinones and cholesterol, which inhibits its entry into the enterocytes, thus not providing the required cholesterol pool for esterification.

Parallel results affirmed [16] a significant reduction in serum total cholesterol and unchanged hepatic total cholesterol concentrations in high cholesterol-fed rats treated with 1 mg/Kg quinazoline derivative (4-amino-2-(4-(bicycle (2, 2, 2) oct-2-ene-5-carbonyl)-1-piperazinyl)-6, 7-dimethoxy-quinazoline). The authors ascribed their findings due to direct inhibition of cholesterol absorption or due to increased biliary excretion of sterol and/or bile acids and the block of cholesterol movement from the liver to the blood.

Also, it is turned out that halogens substitution of quinazolinone has a negative effect on inhibition of hepatic cholesterol esterification displayed by the less pronounced reduction in hepatic free cholesterol and, in contrast the more notable elevation in hepatic cholesterol ester concentrations, compared to quinazolinone (Table 4). Surprisingly, this phenomenon was not observed in case of combined hypercholesterolemia (Table 5). In comparison to bezafibrate, iodoquinazolinone was found to produce a significant more pronounced reduction in serum total cholesterol and cholesterol ester levels. In case of combined hypercholesterolemia, treatment with quinazolinone and its dibromo- and iodo-derivatives produced a significantly favorable effect on serum and hepatic cholesterol profiles than bezafibrate (Tables 3 &5).

In regard to serum lipoproteins profile, the 3 quinazolinones reduced more or less equally serum LDL-C level, whereas serum VLDL-C level was highly reduced by dibromo- and iodoquinazolinone, compared to their reference group. The quinazolinone compounds did not affect significantly serum HDL-C level (Table 2). Taken together, these findings furtherly confirm the inhibitory effect of quinazolinones on cholesterol esterification, as it is well known that LDL particles have a high affinity to bind to cholesterol ester, while it is free cholesterol in case of HDL [35], and that halogens substitution of the quinazolinone increases its triacylglycerol-lowering activity. Consistent with our results, Seki *et al*. [16] referred the decrease of serum total cholesterol level in quinazoline derivative-treated rats to reduction in serum VLDL-C and LDL-C rather than HDL-C levels.

In combined hypercholesterolemia subgroups serum HDL-C and VLDL-C levels were not significantly affected by any of the treatments, whereas serum LDL-C level was significantly decreased by quinazolinones treatment, compared to either bezafibrate or their reference group (Table 3). It is interesting to remark that the more notable reduction in serum LDL-C level in combined than single hypercholesterolemia subgroups might be a sequela of enhanced lipid peroxidation in the former, which converted LDL to modified LDL, including glycosylated, oxidized and small dense LDL particles [36]. Increased liver malondialdehyde (MDA) concentration in the combined hypercholesterolemia subgroups confirms this explanation (Tables 4 &5). However, the non significant changes in hepatic MDA concentration in the different diabetic-hypercholesterolemic subgroups, compared to their reference group, may be due to enhanced lipolysis in diabetes mellitus that exceeded the effects of quinazolinone compounds against LDL peroxidation, which was displayed in non diabetic animals.

Our results are in line with those of Hsun *et al*. [37], who stated that quinazoline derivatives reduce significantly the level of serum LDL-C with respect to the cholesterol reference group. Previous studies documented that serum LDL-C reduction is due to inhibition of hepatic apoB secretion, which is the main apolipoprotein in LDL particles that is recognized by the LDL receptor, and that cholesterol ester availability may be important in regulating apo B secretion. Carr *et al*. [38,39] affirmed that when ACAT is highly inhibited in African green monkey, both apo B and cholesterol ester secretion are reduced, but when ACAT is moderately inhibited cholesterol ester secretion may be reduced without affecting apo B secretion.

Data presented in Tables (2 &3) demonstrate a significant reduction in the atherogenic index (AI) in the quinazolinones-treated subgroups of non diabetic and diabetic hypercholesterolemic rats, compared to their respective reference groups. Non significant differences in the atherogenic index were noticed between the quinazolinones and the bezafibrate. The reduced atherogenic index in both treatments was mainly due to the decrease in serum total cholesterol level rather than elevation in serum HDL-C level.

## Conclusion

1-Subchronic treatments of non diabetic and diabetic-hypercholesterolemic rats with 4(3H)-quinazolinone and its 6, 8-dibromo and 6-iododerivatives (4 weeks) have no significant toxic side effects at the adopted sublethal dose levels (2 mg/Kg).

2-Quinazolinone and its halogenated derivatives possess potential antihyperlipidemic activity in single and combined hypercholesterolemia demonstrated by the dramatic reduction in serum total cholesterol and cholesterol ester levels, and most importantly LDL-C, which is a major risk factor for coronary and cerebral vascular atherosclerosis. The mode of action of quinazolinone compounds is possibly through inhibition of dietary cholesterol absorption and intestinal ACAT activity.

3-Halogen derivatization of quinazolinone confers a triacylglycerol-lowering activity to the parent compound in case of single hypercholesterolemia only.
